# Supplementary material for: Pre-pro is a fast pre-processor for single-particle cryo-EM by enhancing 2D classification
Source: Commun Biol. 2020 Sep 11;3:508. doi: 10.1038/s42003-020-01229-0 (PMC7486923; doi:10.1038/s42003-020-01229-0)
Supplement: Supplementary file 1 — Supplementary Information [file 42003_2020_1229_MOESM1_ESM.pdf]

A Supplementary Notes, Discussion, Method

A.1 Supplementary Materials for Pilot Test

A.1.1 Pilot test on 70S ribosome dataset shows the benefits of using de-noised particles

In this section, we evaluated the effect of de-noising on aligning and grouping similar particle images. There was no iteration involved in this experiment. To perform the test, we chose a widely used dataset of E. coli 70S ribosome [1–7] downloaded from RELION classification benchmark dataset as described in the Methods. This dataset contains a total of 10,000 particles in a box of 130×130 pixels. In this study, we only use the first 5,000 particles, which have a translation factor (EF-G) bound on the ribosome. For the sake of simplicity, we term this set "70S ribosome" throughout this article.

We first generated a de-noised set of the 5,000 70S ribosome particles using the 2SDR de-noising method. Some of the de-noised particles are displayed in the illustration of the workflow Figure 1(b). For the control experiment, we randomly picked five particles as references (column (a) of Supplementary Figure 1). To each reference, we searched within the set for most resembling particles, for which we used a Matlab program re-coded from FRM2D [8], a fast alignment algorithm, to facilitate the alignment during the search. For a resembling particle, we recorded the best alignment parameters −the rotation angles and translation shifts in x-and-y-direction. For each reference we selected the 20 most resembling particles. By applying the alignment parameters to each of the 20 particles, we generated the average. The results are displayed in column (b) of Supplementary Figure 1. We then repeated this procedure with the de-noised set as we did in the control experiment. In brief, the de-noised particles are to replace the original particles except in the end the alignment parameters are again applied to the original particles for calculating the aligned average, as shown in column (c) in Supplementary Figure 1. It can be observed that the average from the 20 most resembling images found using the de-noising procedure (column (c) in Supplementary Figure 1) has more structural details than that without the de-noising (column (b) in Supplementary Figure 1), suggesting that de-noising do help the searching and aligning of the particles. Importantly, these averages (column (c) in Supplementary Figure 1) are more consistent with the corresponding projections of the 70S ribosome, shown in column (d) in Supplementary Figure 1.

A.1.2 Pilot test on the 70S ribosome simulation dataset quantifies the rate of true positives in grouping alike particles

As the matching set composed by the 20 most resembling particles found with the de-noising do not overlap well with that of no de-noising, we furthered a study using simulated data to measure the occurrence of true positives. The simulated noisy images of 70S ribosome were then prepared as follows. First, the 3D structure was calculated from the 5,000 experimental images using CryoSparc. Then a total of 50 distinct 2D images with 130×130 pixels were generated by projecting the 3D structure of 70S ribosome in equally spaced (angle-wise) orientations. Secondly, 5,000 images were generated from these 50 projections by making 100 copies for each projection. For each projection, the 100 copies from $i = 1$ to $i = 100$ are uniformly rotated with $3.6 \times i$ degree. Thirdly, each image was then convoluted with the electron microscopy contrast transfer function randomly sampled from a set of 50 CTF values. [3] Finally, i.i.d. Gaussian noise $N\left(0, \sigma^2\right)$ was added such that the signal-to-noise ratio (SNR) equals to 0.01. To measure the occurrence of grouping true alike particles, we repeated the above-mentioned procedure for 10 times. For this simulation dataset the average number of the correct particles among the top 100 is found to be 3.5 and 11.2 for the group without and with the 2SDR de-noising, converted to the frequency of true positive 3.5% and 11.2% respectively (Supplementary Figure 2). With the SNR increased to 0.05, the corresponding frequencies increase to 61.5% and 94.4% (Supplementary Figure 3). With the defocus lowered to the range of 1 to 1.5 mm, these figures drop to 51.3% and 93.7% (Supplementary Figure 4). These results show that the 2SDR de-noising helps grouping true alike particle especially when the SNR or defocus is lower.

A.2 Supplementary Materials for RELION Analysis

In this section, we document the statistics output from the classification programs, by which we can utilize to evaluate the performance in an objective way. As these statistics are associated with each class, we use the histogram to plot the number of classes with respect to a statistics quantity.

For comparing RELION with P-RELION, two statistics that represent the alignment errors [9], ”rlnAccuracyRotations” and ”rlnAccuracyTranslations” are used. In brief, for each iteration, the program will select a random subset of images from each class and assume the orientations of these selected images in the previous iteration are correct. Then, the program will modify the orientations of each image in small steps until the ratio of posterior probability between current modified orientation and

[3] Here, the defocus is randomly sampled from 1.5 um to 2 um and the astigmatism angle is from 0.2 to 1.4 radian. The electron beam accelerating voltage was set to be 300KeV with spherical aberration Cs = 2mm, amplitude contrast=0.07 and pixel size is 2.82Å.

true orientation is smaller than 0.01. The averages of the corresponding rotation and translation steps for each case are recorded in the "rlnAccuracyRotaions" and "rlnAccuracyTranslations". One can expect that the value of steps will be small if the orientations can be distinguished reliably. These values can thus be considered as the alignment errors of RELION while the lower is the better. These values are used as the guideline for selecting good class averages in this work. [4]

A.3 Supplementary Materials for ISAC Analysis

For ISAC, there are also two statistics associated with each class as defined in [10]. They are pixel error and the class size, which can be used to compare ISAC with P-ISAC. [5] First, the pixel error is the statistics computed using the alignment parameters from independent rounds of within-class alignment, which is so called the "stability test" and it will be lowered if the class becomes more homogeneous. From Supplementary Figure 8, we can see that with P-ISAC, the pixel error becomes lowered and the number of classes with small pixel error increases. Secondly, the class size indicates how many images pass the above stability test; it again represents the performance of the clustering step and will be larger if a class is more homogeneous. From Supplementary Figure 9, we can see that the class with small size becomes fewer by P-ISAC as compared to ISAC.

A.4 Supplementary Materials for CL2D Analysis

We further demonstrate our method on a popular classification algorithm called CL2D. CL2D [2] belongs to the category of classification algorithms that is based on K-means. It develops an approach based on divisive hierarchical clustering and adopts a new probabilistic similarity metric based on correntropy to reduce the influence of noise and obviate the phenomenon of unbalanced classes. When preprocessing is added to CL2D, we abbreviate the procedure as P-CL2D in the same way as in the main article.

The classification results of CL2D and P-CL2D are shown in Supplementary Figure 18. Since it is a divisive hierarchical clustering algorithm, the class number is set to 64, 64, 512 and 128 (power of 2) for 70S ribosome, beta-galactosidase, 80S ribosome and TRPV1, respectively. For the 80S ribosome and TRPV1, we down-sampled the images by 4X and 2X to shorten the time spent on CL2D. The class averages are sorted according to their class size as in the experiment of RELION. The classification results are shown in Supplementary Figure 18, 21 and 22. More features of particle are observed from

[4] Selection by expert is still needed. From Supplementary Figure 5, we can see that P-RELION usually increases the number of stable class and thus the total number of classes we choose will also increase.

[5] Since the particles remain in the pool will be different after the first main iteration (the target number of classes will thus be different in the following iterations), we plot the statistics with respect to the number of classes for the first main iteration.

the P-CL2D classification results. For instance, the number of off-centered class averages is reduced in the case of beta-galactosidase dataset, as shown in Supplementary Figure 18(c) and (d). The 3D initial model calculated from the class averages from P-CL2D is also better than the one from CL2D as depicted in Supplementary Figure 20. Besides the quality of class averages, the classification time of CL2D and P-CL2D are also recorded in Supplementary Table 1. The classification time is roughly the same due to the number of iterations is fixed in the CL2D implementation. However, when we calculate the number of images that change class assignment during each iteration, which is regarded as one of the convergence criterion in the CL2D algorithm [2], faster convergence speed is observed for P-CL2D as shown in the Supplementary Figure 19. Finally, the resolution determined by FSC is recorded in Supplementary Table 2. We can perceive that our method, again, helps to improve the final resolution.

B Supplementary Figures

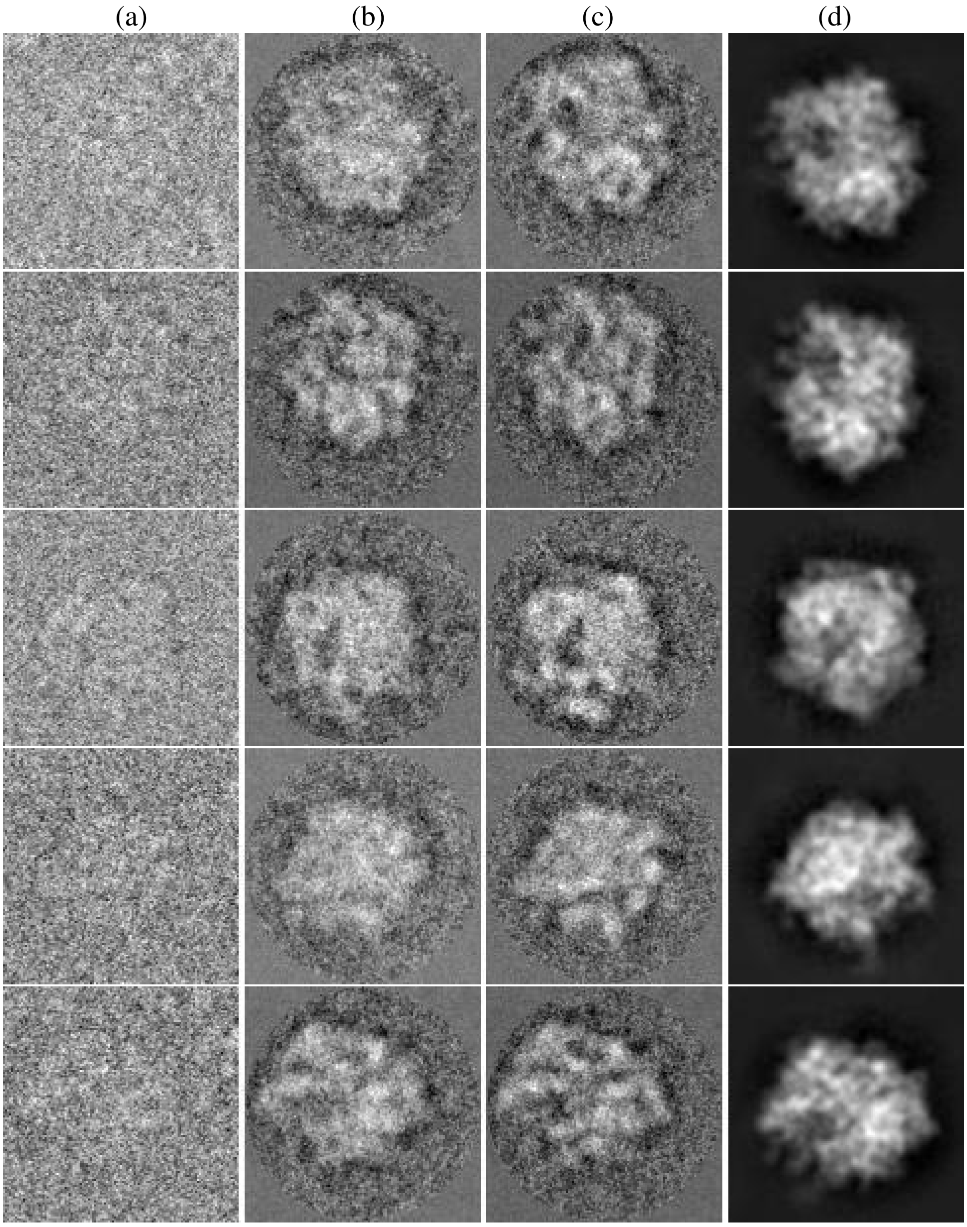

Supplementary Figure 1: Pilot tests of the effect by 2SDR on grouping and aligning 70S ribosome particles. Column (a) shows five cryo-EM images of 70S ribosome randomly picked from the 5,000 cryo-EM images and used as references; note that they will not be centered but will be masked by a circle for the purpose of rotation in the alignment process. Column (b) shows an aligned average of the 20 particles that most resemble their reference in (a). Column (c) shows the aligned average of the 20 particles whose de-noised images most resemble their de-noised reference (the de-noised images are not shown). Column (d) shows the projections of the structure of 70S ribosome that correspond to the views in Column (a).

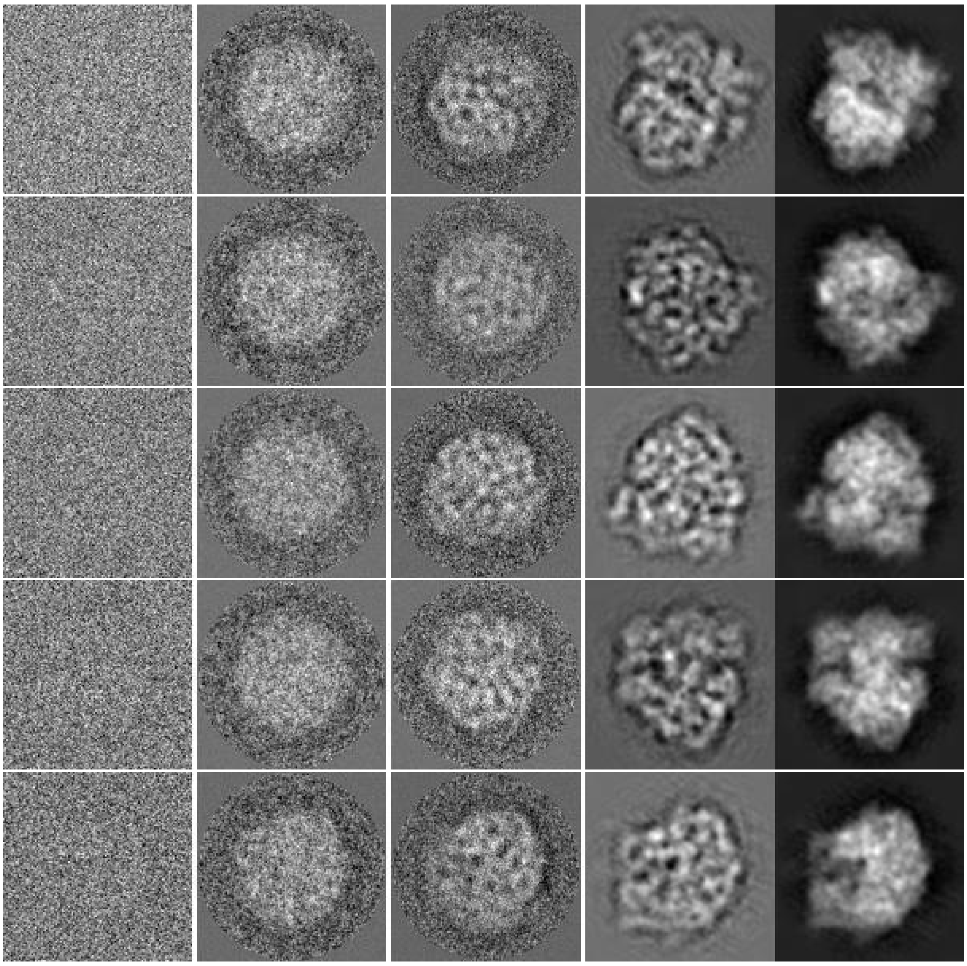

Supplementary Figure 2: Pilot Test with Simulation Data of 70S ribosome Where SNR = 0.01 with defocus randomly sampled between 1.5um to 2.0um. From left to right are Column (1) to (5). Column (1) represents five simulated cryo-EM images used as the references, which are produced from the projection images in Column (5) with the application of CTF and addition of noise; Column (2) represents the average from the 100 particles that most resemble the reference in (1); Column (3) the same as (2) but the average is from the 100 most resembling particles found after 2SDR de-noising is applied. Column (4) Noise-free images of Column (1) to show the ground truth, which are produced from the projection images in Column (5) by application of CTF. (5) Five projection images of 70S ribosome.

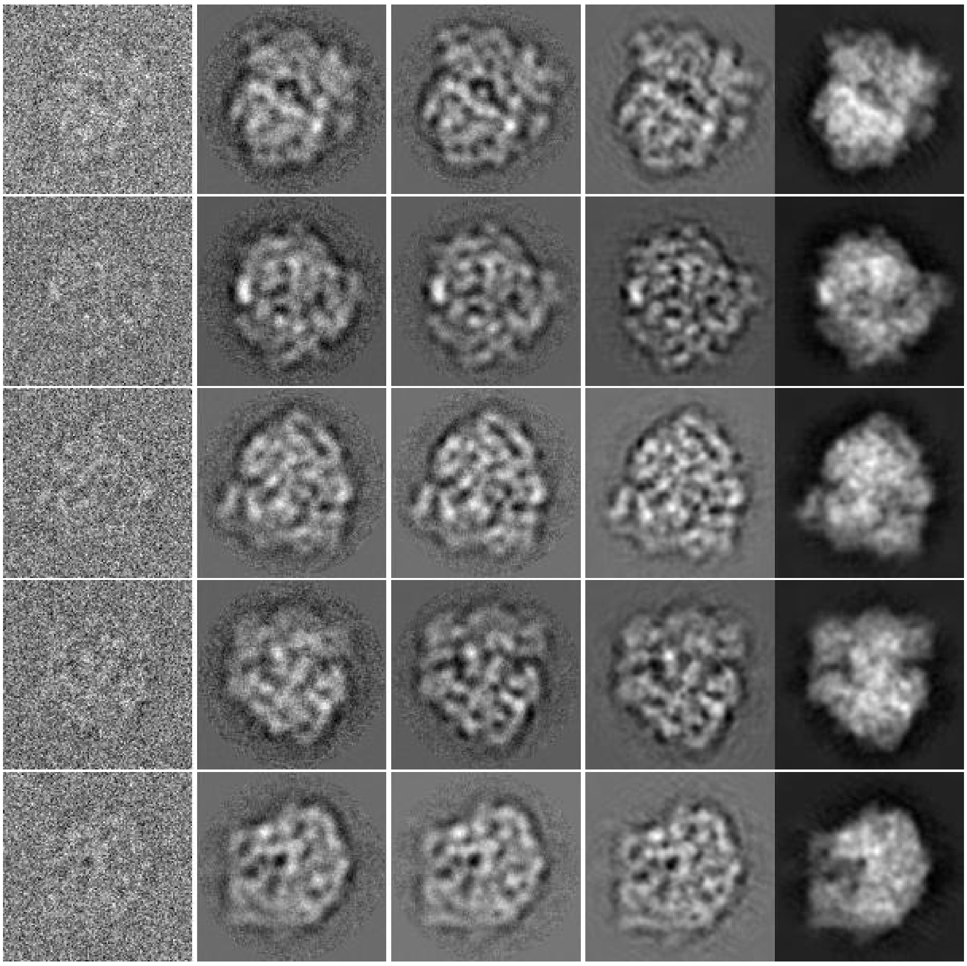

Supplementary Figure 3: Pilot Test with Simulation Data of 70S ribosome Where SNR = 0.05 with defocus randomly sampled between 1.5um to 2.0um. From left to right are Column (1) to (5). Column (1) represents five simulated cryo-EM images used as the references, which are produced from the projection images in Column (5) with the application of CTF and addition of noise; Column (2) represents the average from the 100 particles that most resemble the reference in (1); Column (3) the same as (2) but the average is from the 100 most resembling particles found after 2SDR de-noising is applied. Column (4) Noise-free images of Column (1) to show the ground truth, which are produced from the projection images in Column (5) by application of CTF. (5) Five projection images of 70S ribosome.

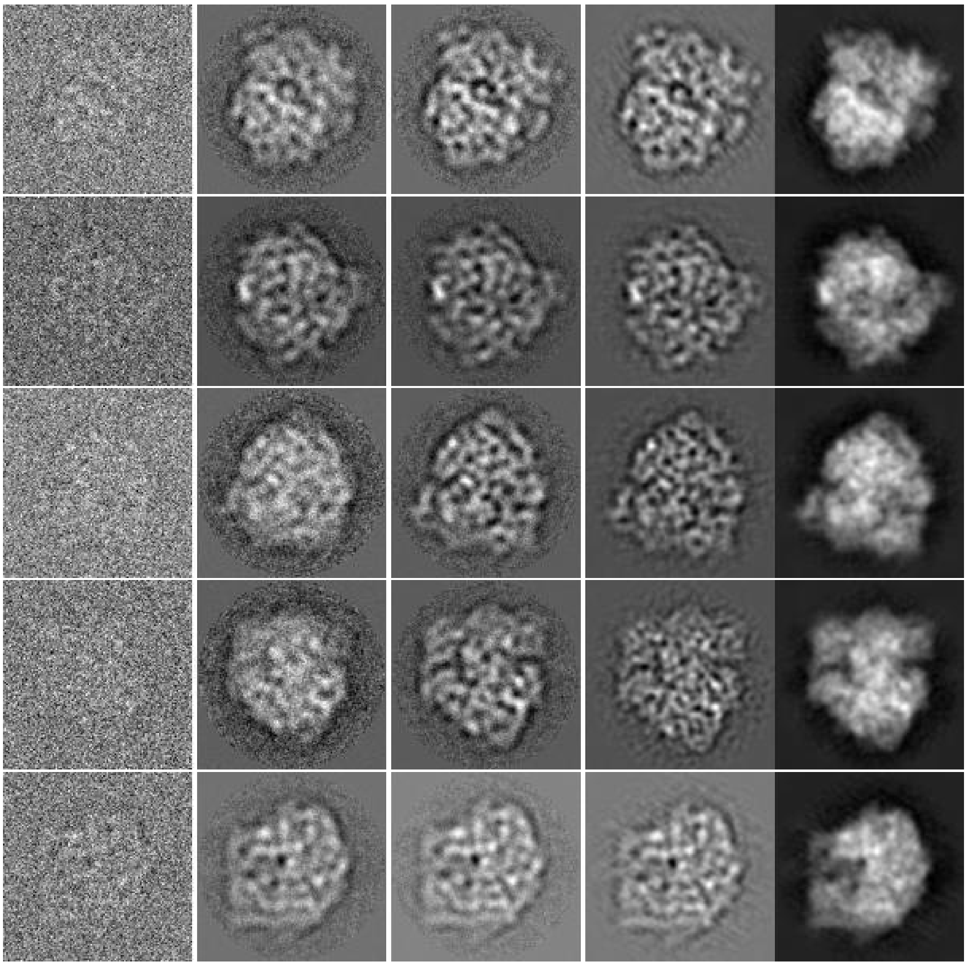

Supplementary Figure 4: Pilot Test with Simulation Data of 70S ribosome Where SNR = 0.05 with defocus randomly sampled between 1.0um to 1.5um. From left to right are Column (1) to (5). Column (1) represents five simulated cryo-EM images used as the references, which are produced from the projection images in Column (5) with the application of CTF and addition of noise; Column (2) represents the average from the 100 particles that most resemble the reference in (1); Column (3) the same as (2) but the average is from the 100 most resembling particles found after 2SDR de-noising is applied. Column (4) Noise-free images of Column (1) to show the ground truth, which are produced from the projection images in Column (5) by application of CTF. (5) Five projection images of 70S ribosome.

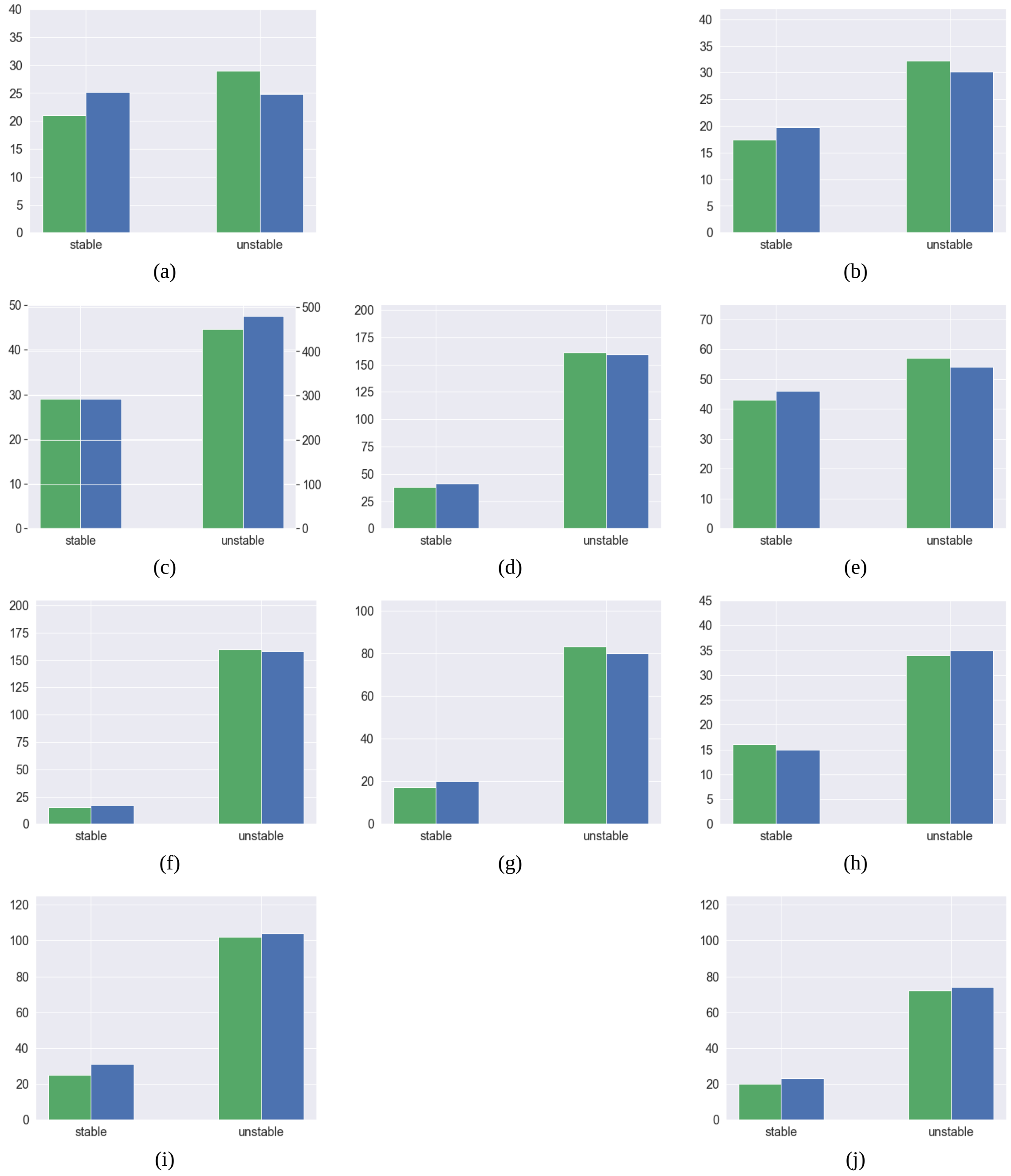

Supplementary Figure 5: RELION Statistics. We seperate the class according to the "rlnAccuracyRotations" and "rlnAccuracyTranslations". We put the class into the unstable class bin if the statistics are 999 which means the alignment error is large. The remaining classes are put into the bin of stable class. Results of RELION and P-RELION are represented as green and blue bars, respectively. The unit of Y-axis is the number of classes. (a) Results of 70S ribosome. (b) Results for beta-galactosidase. (c), (d) and (e) Results for 80S ribosome with 520, 200 and 100 class, respectively. (f), (g) and (h) Results for TRPV1 with 175, 100 and 50 class, respectively. (i) Results for NC-TRPV1 with 200 class. (j) Results for NanoD-TRPV1 with 100 class. Note that there are two scales in the Y-axis for 80S ribosome with 520 class.

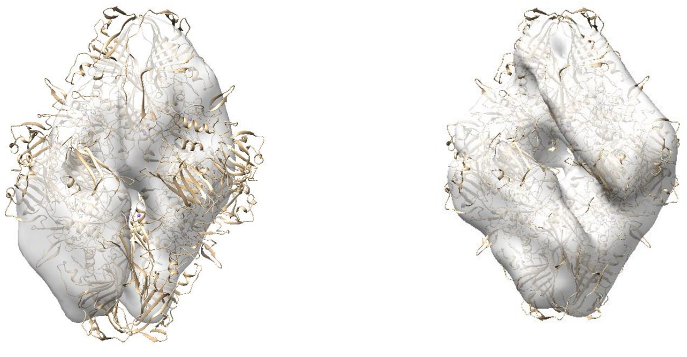

(a) (b)

Supplementary Figure 6: Rigid Body Fitting. With Chimera, we fit an atomic model to the two initial models generated by RELION and P-RELION class averages. In (a), the atomic model is fitted to the initial model generated from the class averages produced by RELION. In (b), the atomic model is fitted to the initial model generated from the class averages produced by P-RELION.

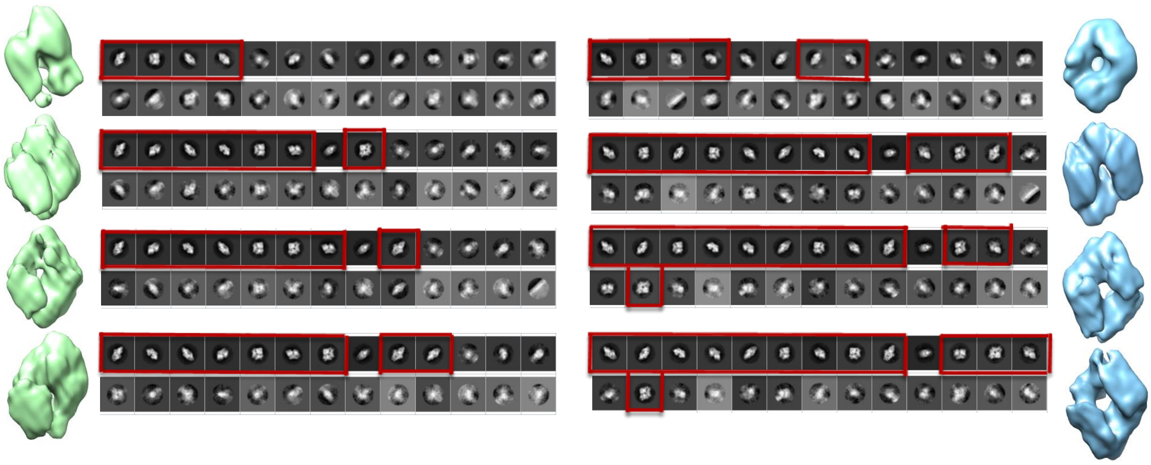

Supplementary Figure 7: The behavior of RELION and P-RELION in different iterations. Four rows show the top classes from iteration 5,10, 15 and 25, respectively. The left part are the results of RELION, and the right part are the results of P-RELION. The initial models generated by the selected class averages are shown along with the classification results from different iterations.

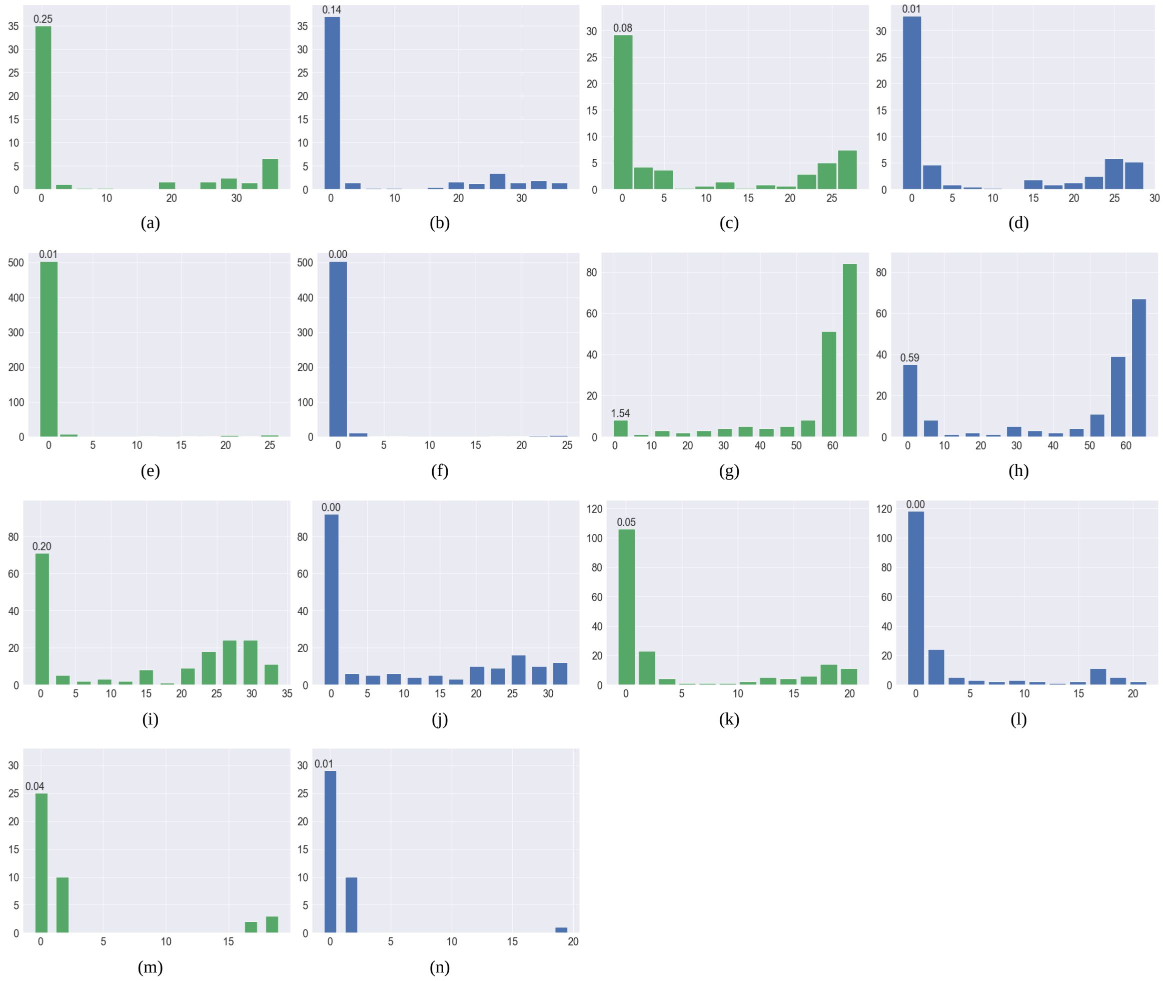

Supplementary Figure 8: ISAC Pixel Error Statistics. The first and third columns are the results associated with ISAC, while the second and fourth columns are the results for P-ISAC. The unit of X-axis stands for pixel error and Y-axis is for the number of classes. (a)(b) Results for 70S ribosome. (c)(d) Results for beta-galactosidase. (e),(f) Results for 80S ribosome. (g)(h), (i)(j) and (k)(l) Results for TRPV1 with no down-sampling, down-sampling with 2X and down-sampling with 3X, respectively. (m)(n) Results for NC-TRPV1. The exact pixel error of the first bin is labeled on top of the bar.

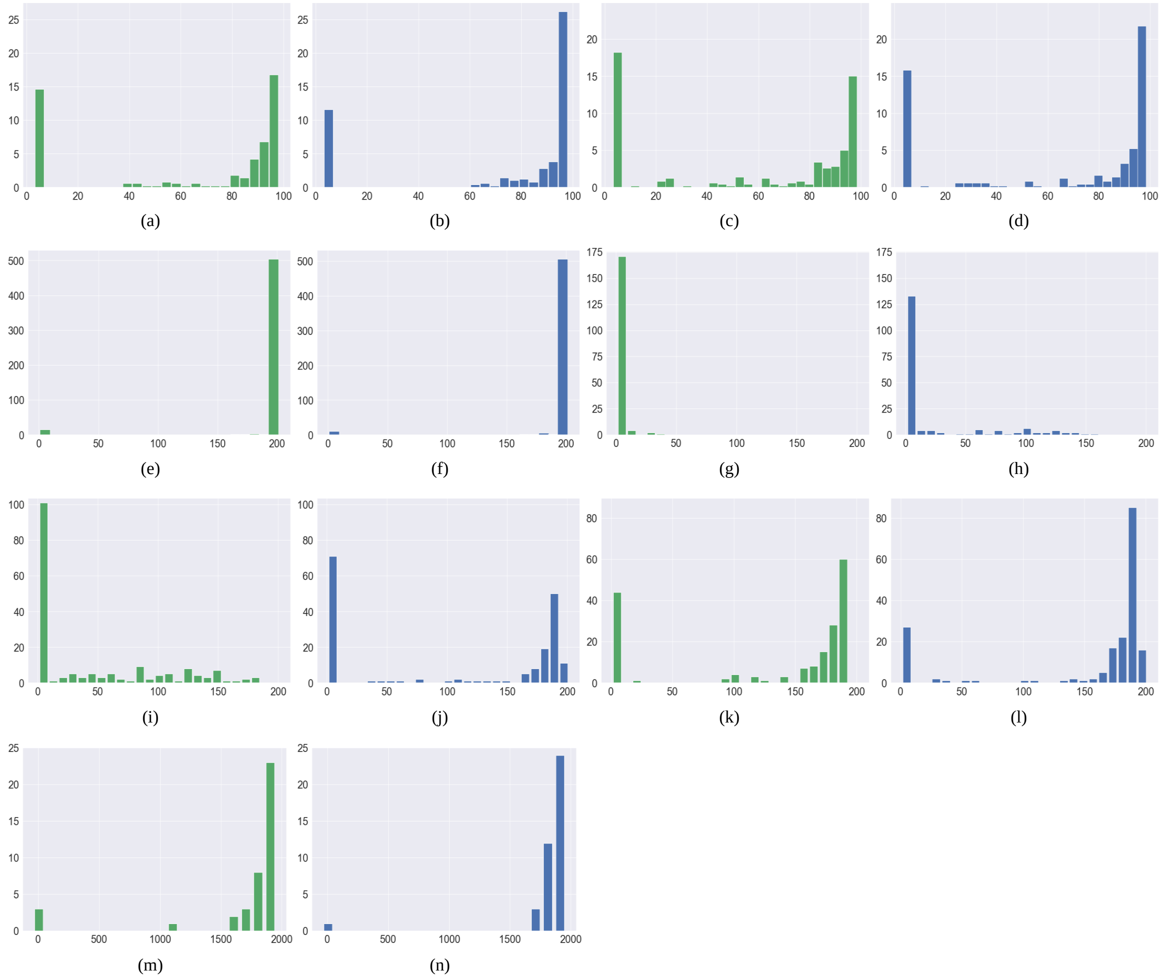

Supplementary Figure 9: ISAC Class Occupancy Statistics. The first and third columns, colored in green, are the results associated with ISAC, while the second and fourth columns, colored in blue are the results for P-ISAC. The unit of X-axis stands for the class size and Y-axis is for the number of classes. (a)(b) Results for 70S ribosome. (c)(d) Results for beta-galactosidase. (e)(f) Results for 80S ribosome.(g)(h), (i)(j) and (k)(l) Results for TRPV1 with no down-sampling, 2X down-sampling and 3X sampling, respectively. (m)(n) Results for NC-TRPV1.

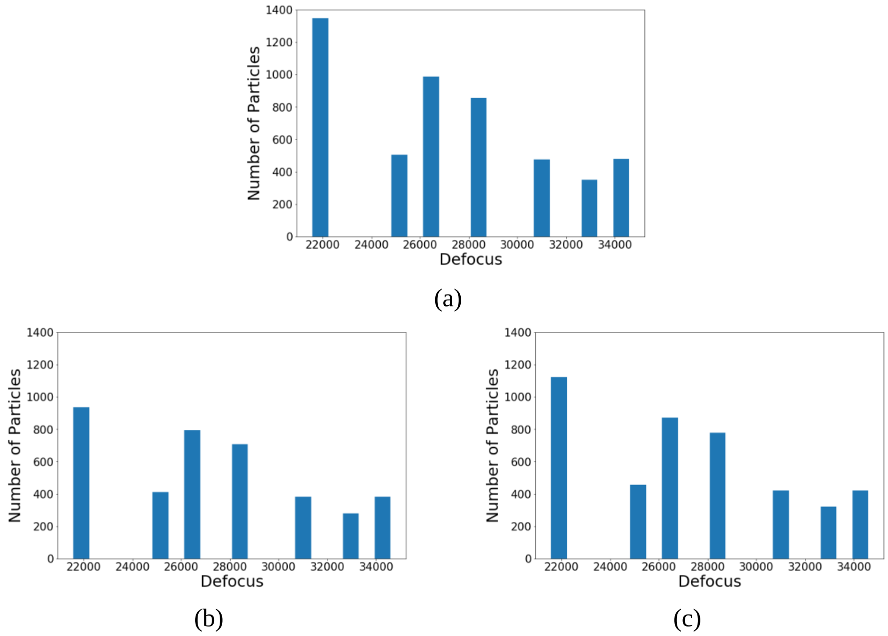

Supplementary Figure 10: Histogram of particle's defocus values in Å (10^{-4}um): number of particles vs defocus values. (a) Original 70S ribosome dataset. (b) Curated subset after performing ISAC. (c) Curated subset after performing P-ISAC.

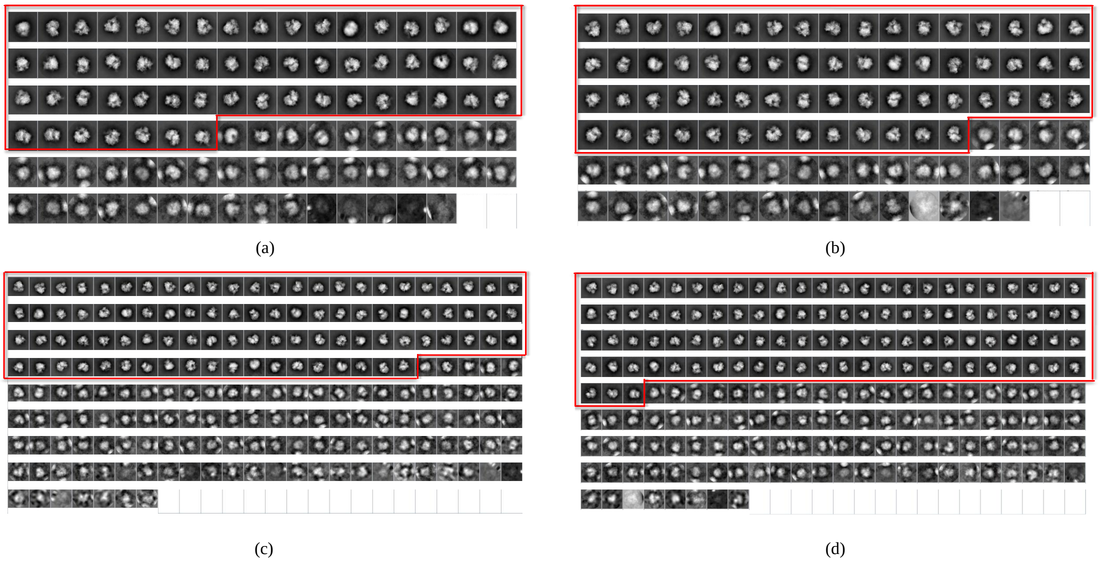

Supplementary Figure 11: Classification Results of (a)(c) RELION and of (b)(d) P-RELION on 80S ribosome with the class size set to 100 and 200, respectively. The selected class averages are boxed in red.

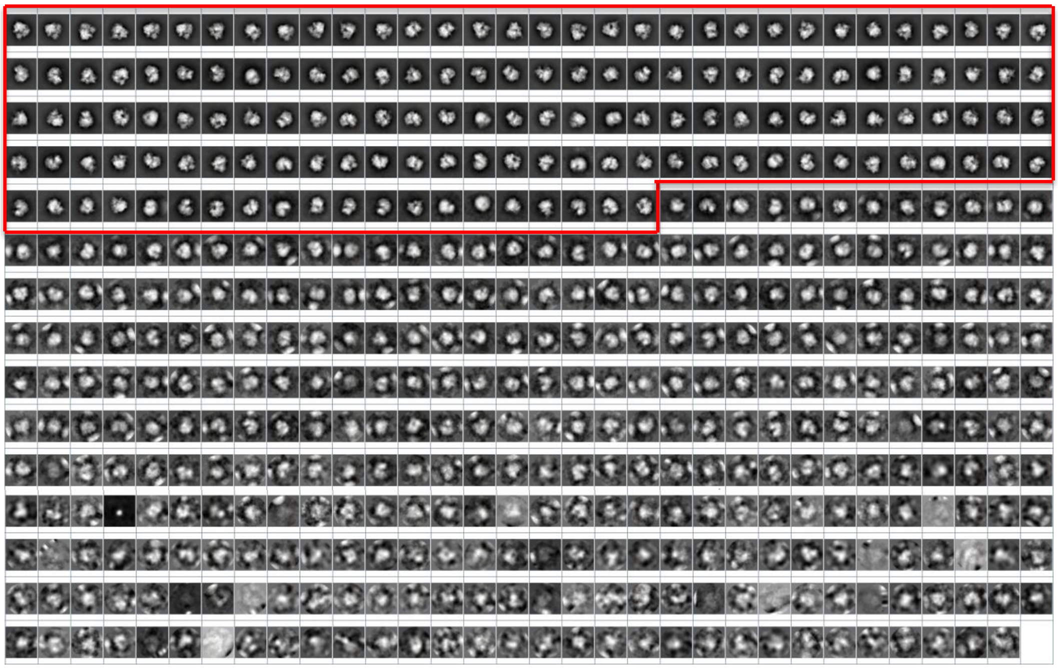

(a)

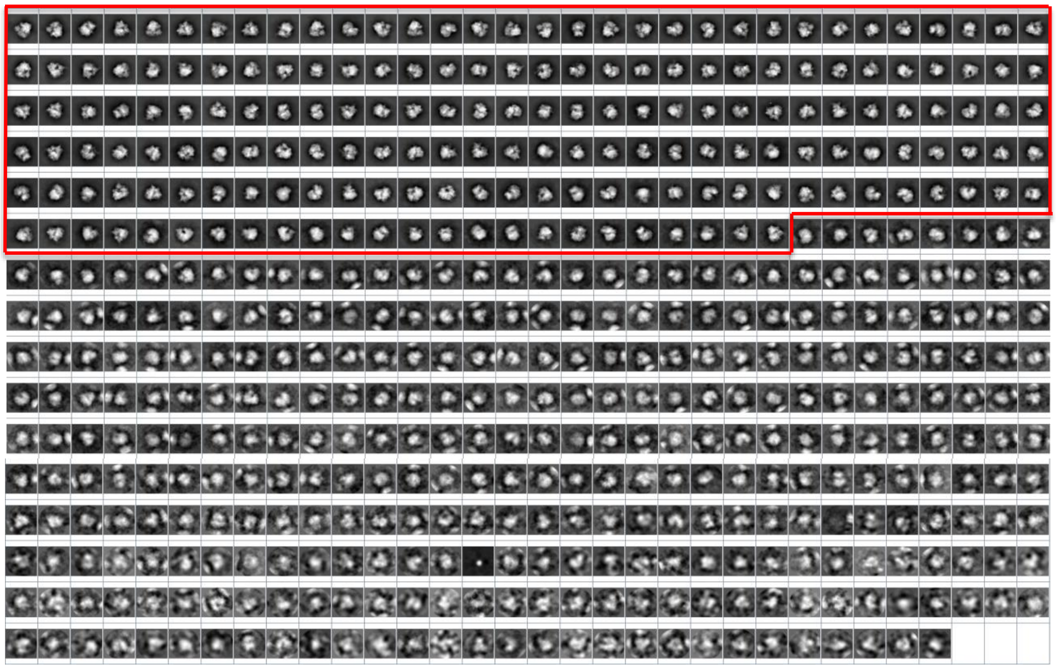

(b)

Supplementary Figure 12: Classification Results of (a) RELION and (b) P-RELION on 80S ribosome with the class size set to 520. The selected class averages are boxed in red.

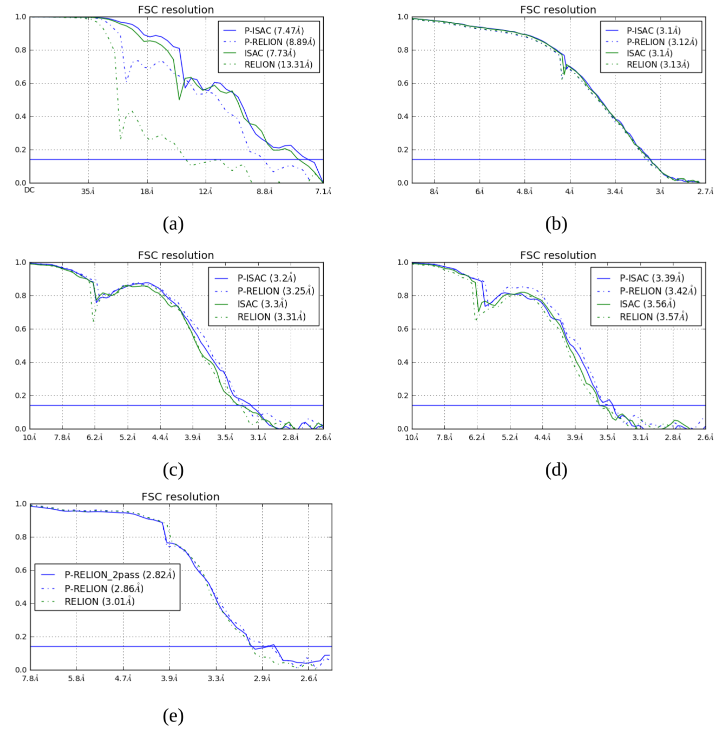

Supplementary Figure 13: Best Standard FSC Curves for Describing the Overall Resolution of a 3D Reconstruction: (a) beta-galactosidase, (b) 80S ribosome, (c)TRPV1, (d) NC-TRPV1 and (e) NanoD-TRPV1.

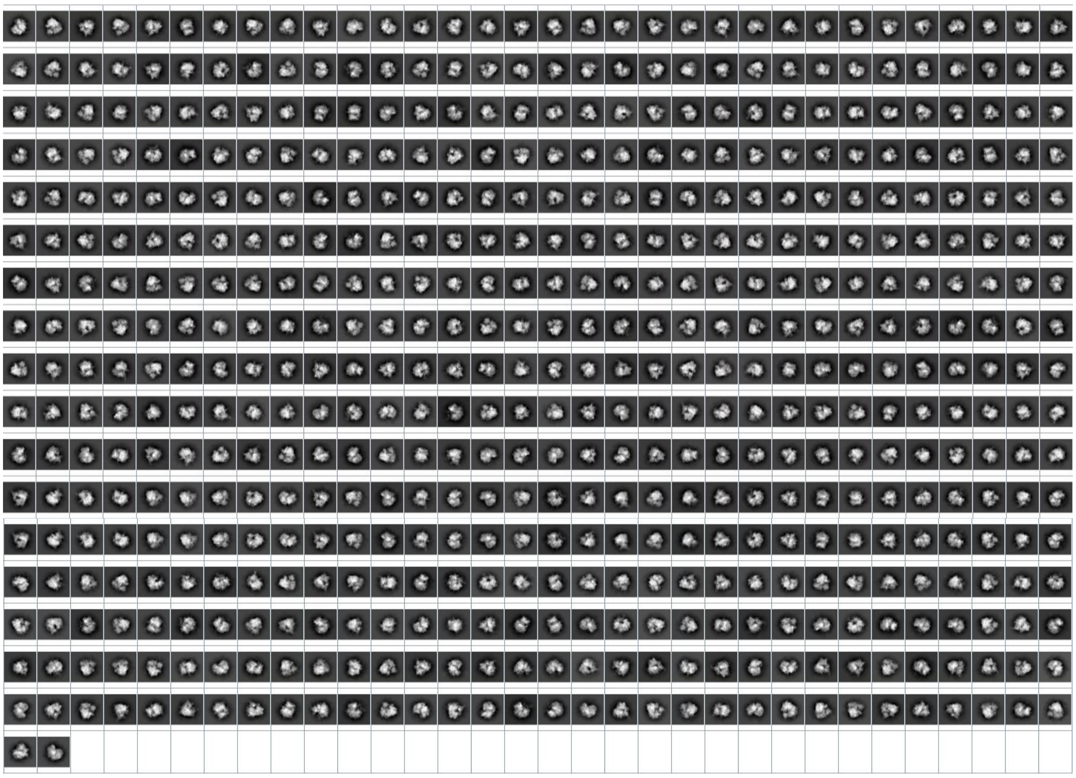

(a)

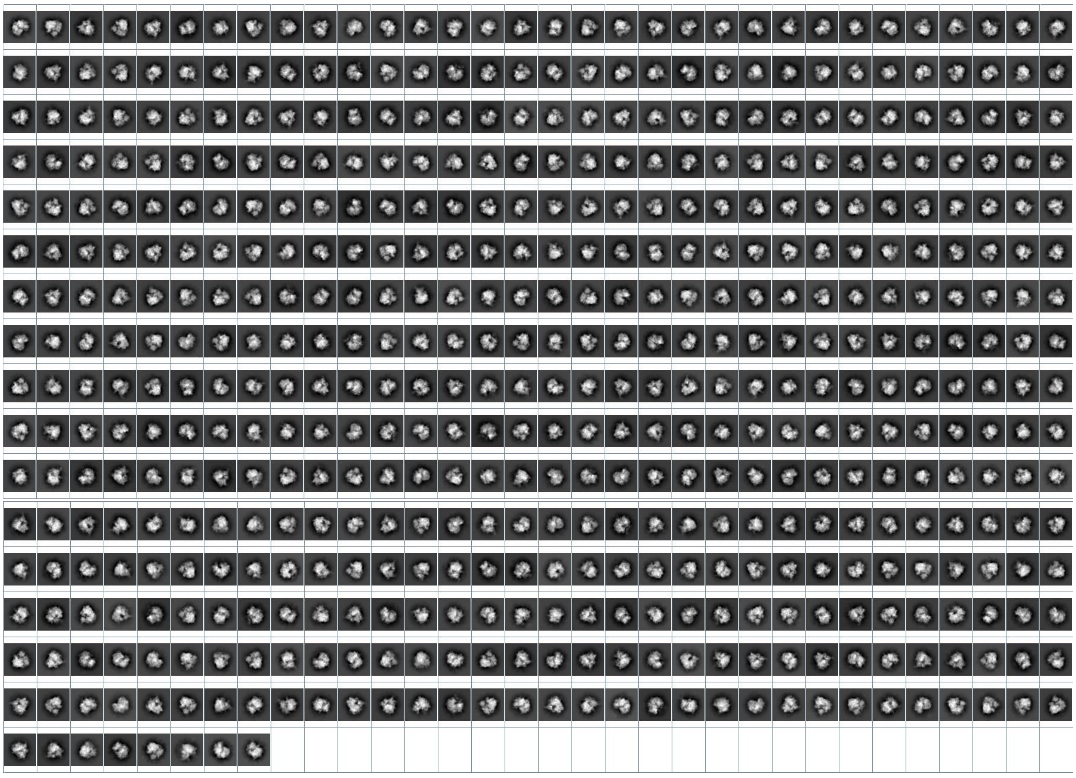

(b)

Supplementary Figure 14: Classification 80S ribosome with ISAC. (a) 514 classes produced by ISAC and (b) 520 classes produced by P-ISAC.

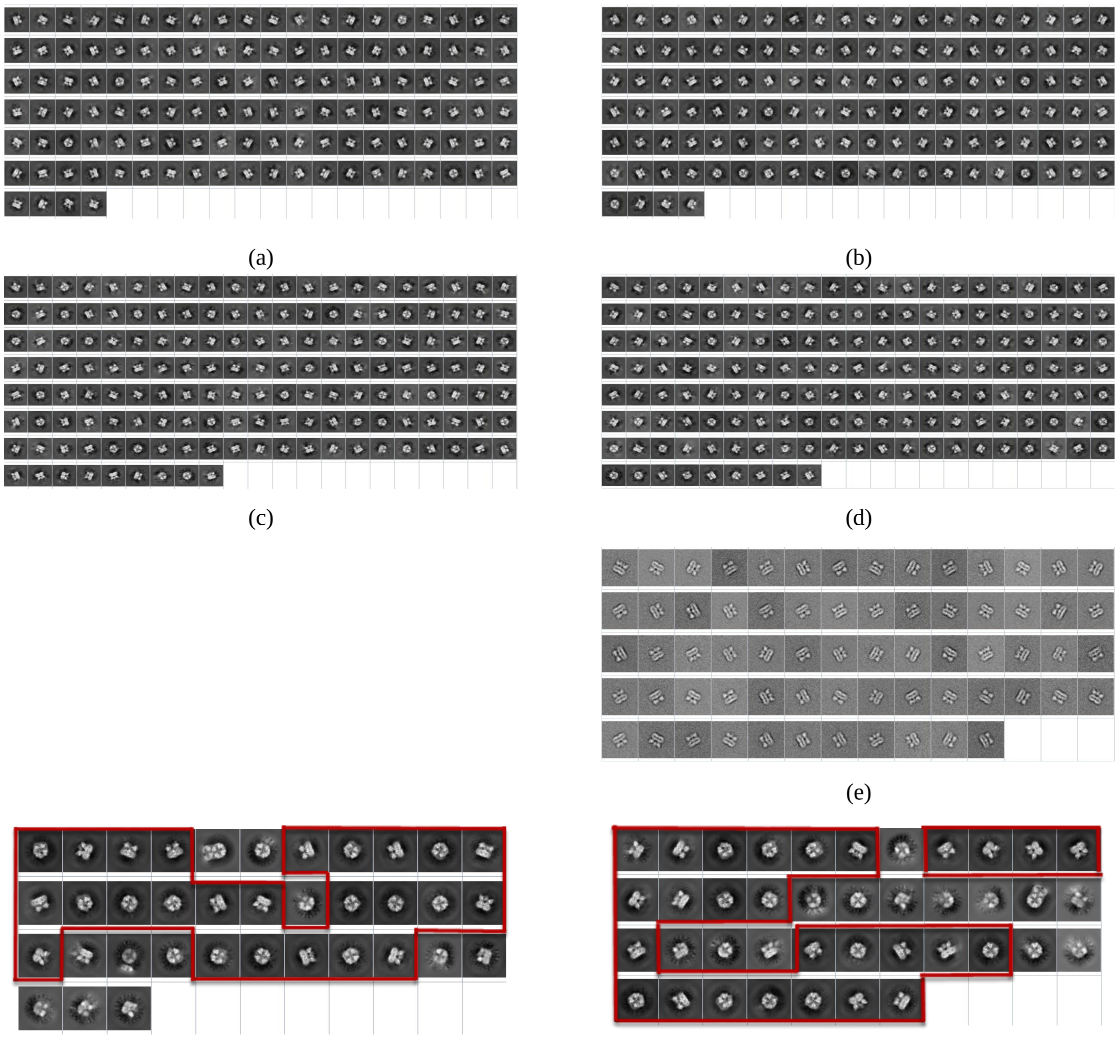

(a) (b) (c) (d) (e) (f) (g)

Supplementary Figure 15: Classification Results of TRPV1 using ISAC. (a) and (c) ISAC with down-sampling 2X and 3X. (b),(d) and (e) P-ISAC with sampling 2X, 3X and no down-sampling. Note that ISAC is not able to generate class averages without down-sampling. On the other hand, (f) and (g) corresponds to classification results of ISAC and P-ISAC on NC-TRPV1. The selected class averages are boxed in red.

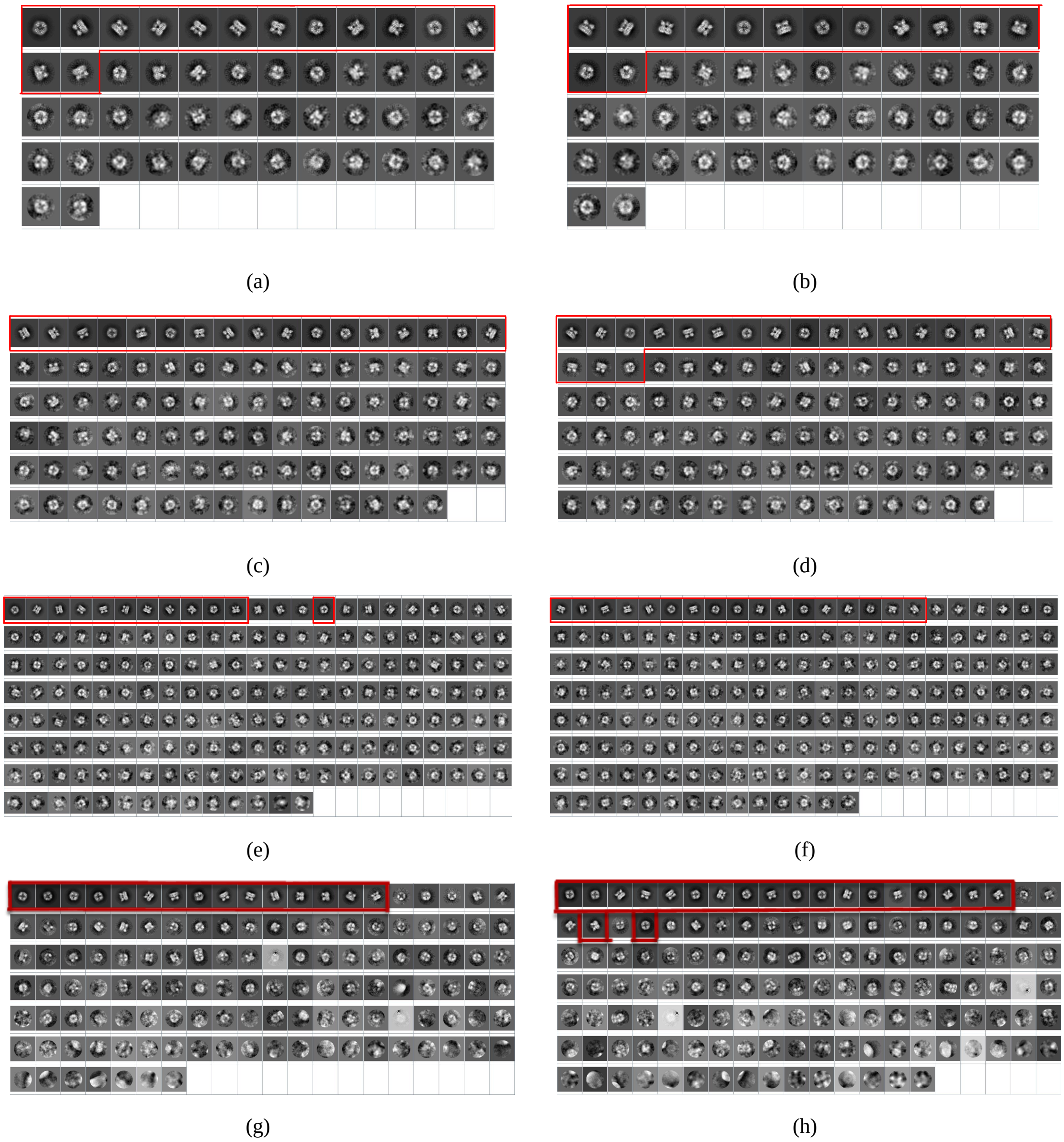

Supplementary Figure 16: Classification Results of RELION on TRPV1. (a),(c) and (e) correspond to the class size set to 50, 100, and 175, respectively. (b),(d) and (f) are from P-RELION with the same setting as RELION. On the other hand, (g) and (h) corresponds to classification results of RELION and P-RELION on NC-TRPV1. The selected class averages are boxed in red.

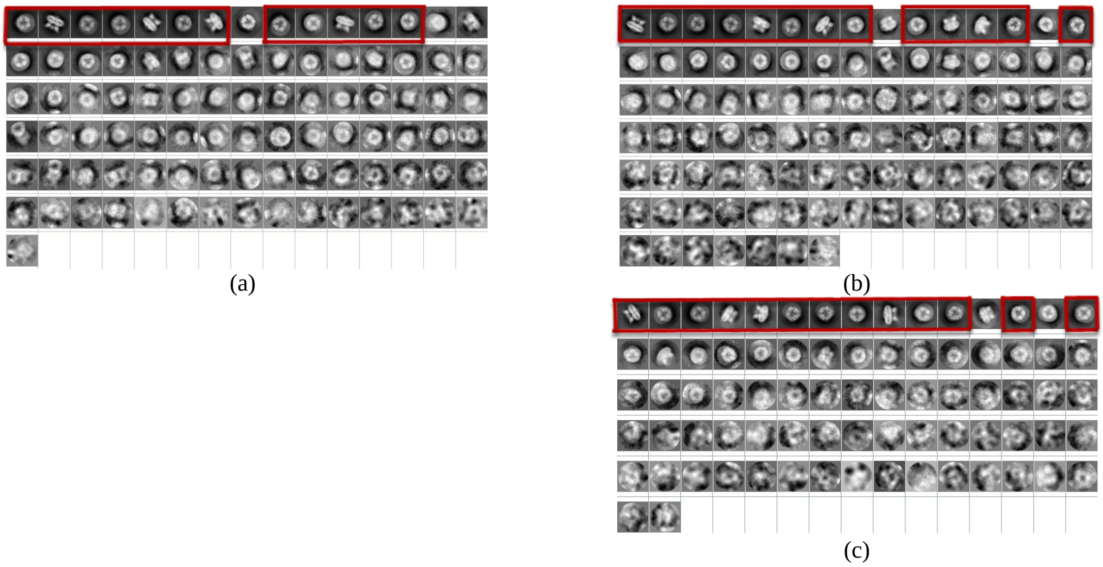

Supplementary Figure 17: Classification Results of NanoD-TRPV1 using RELION. (a) RELION with 100 Classes. (b) P-RELION with 100 Classes. (c) Second Pass of P-RELION with 100 classes. The selected class averages are boxed in red.

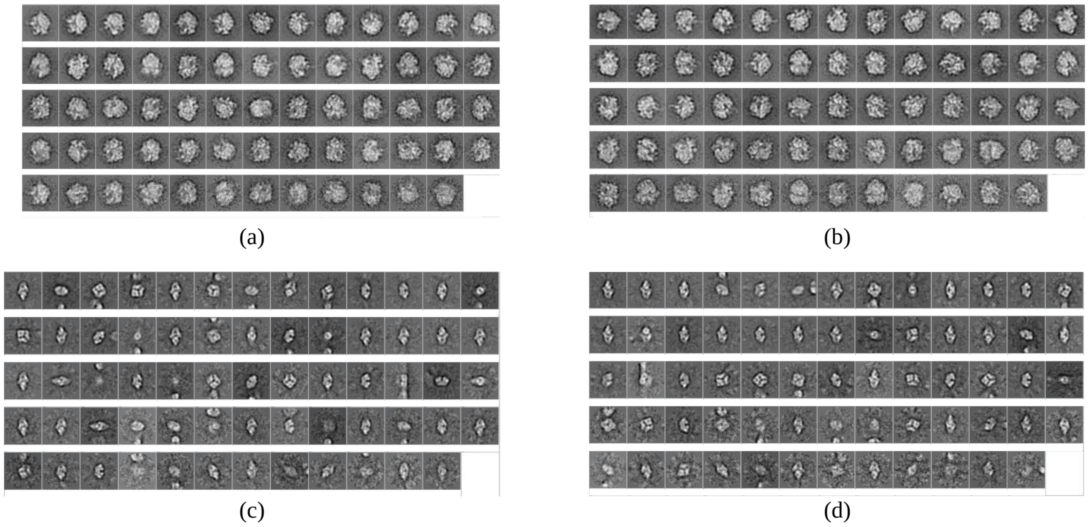

Supplementary Figure 18: Classification Results of (a) CL2D and (b) P-CL2D on 70S ribosome. Classification results of (c) CL2D and (d) P-CL2D on beta-galactosidase.

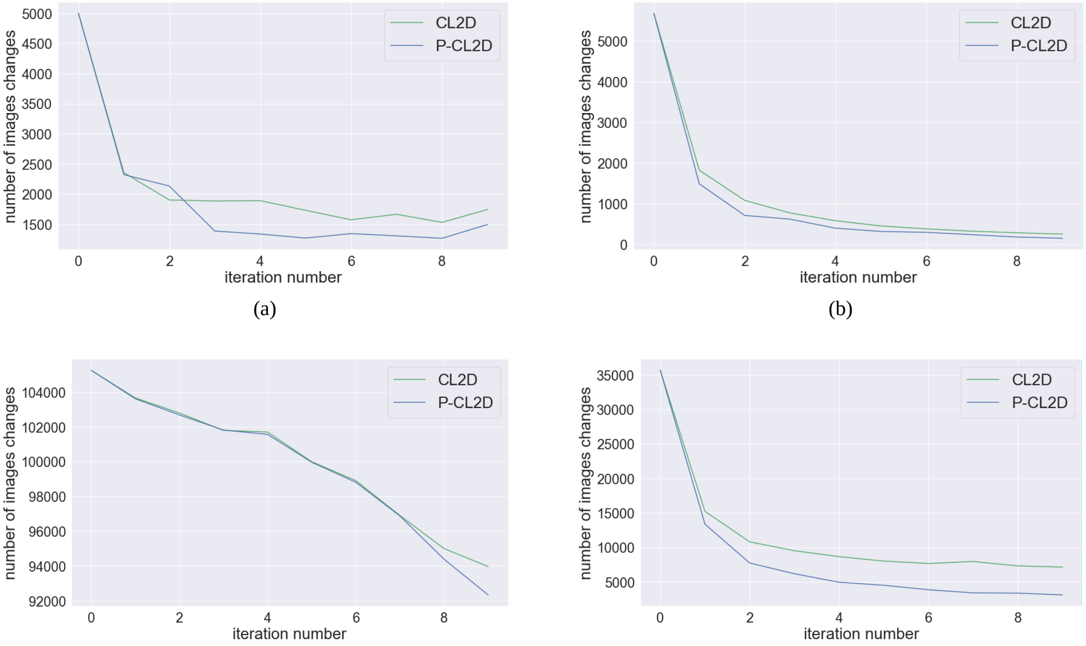

(a) (b) (c) (d)

Supplementary Figure 19: Convergence Speed of CL2D and P-CL2D on (a) 70S ribosome, (b) beta-galactosidase, (c) 80S ribosome and (d) TRPV1 dataset.

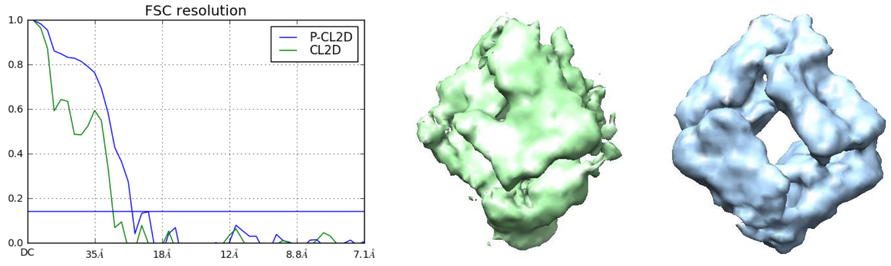

(a) (b) (c)

Supplementary Figure 20: The 3D initial models generated by 2D classification averages of beta-galactosidase dataset. (a) FSC between the reference map and ab initio model generated by CL2D and P-CL2D. (b) and (c) are the initial models generated by 2D classification averages outputted from CL2D and P-CL2D.

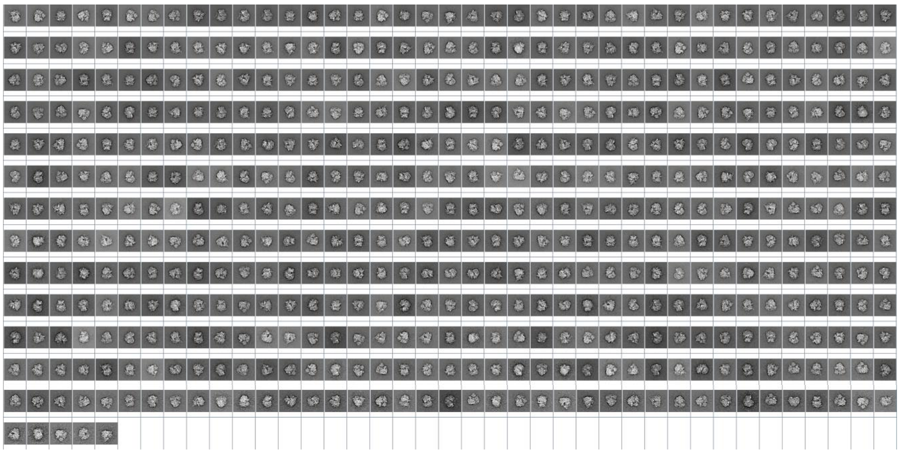

(a)

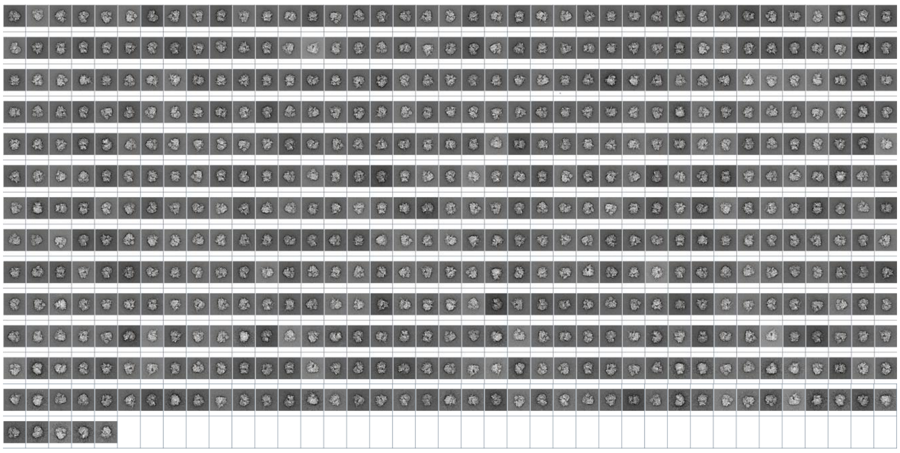

(b)

Supplementary Figure 21: Classification Results of (a) CL2D and (b) P-CL2D on 80S ribosome.

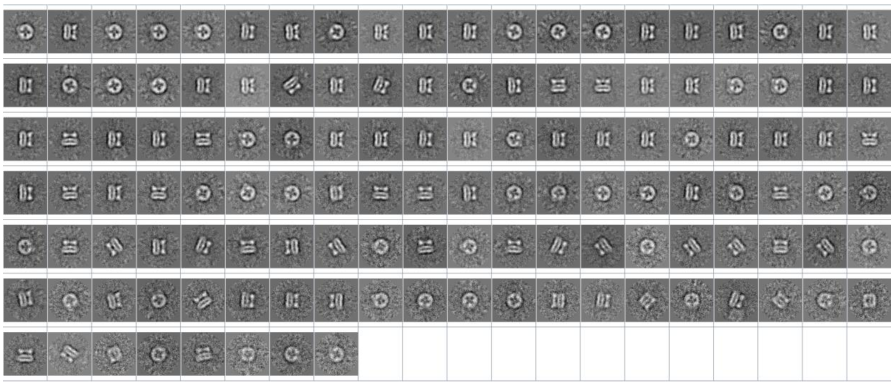

(a)

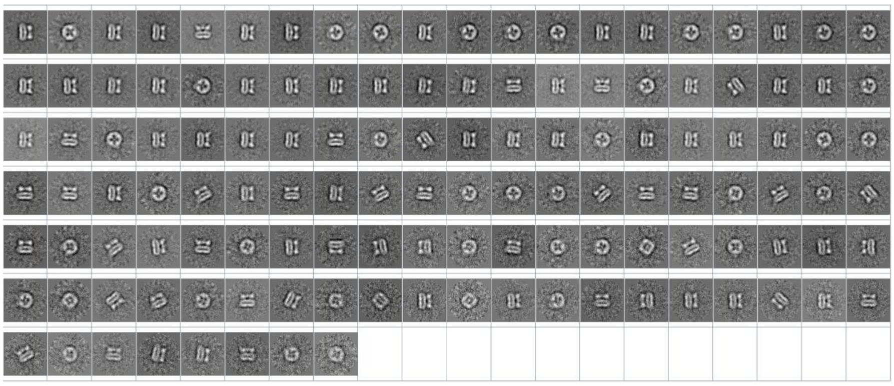

(b)

Supplementary Figure 22: Classification Results of (a) CL2D and (b) P-CL2D on TRPV1.

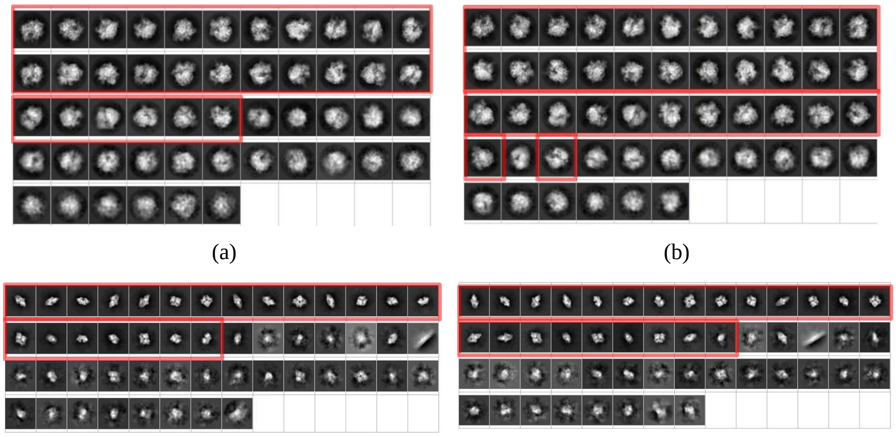

(a) (b)

(c) (d)

Supplementary Figure 23: Classification Results of (a) CryoSparc and (b) P-CryoSparc on 70S ribosome dataset. (c) CryoSparc and (d) P-CryoSparc on beta-galactosidase dataset.

C Supplementary Tables

Supplementary Table 1: The classification time of CL2D and P-CL2D on four datasets

Algorithm	Dataset	Number of Iterations	Number of MPI/GPU	Execution Time (Hour) (Original/with Preprocessing)
CL2D	70S ribosome	10	10/0	3.06/3.07
	Beta-galactosidase	10	10/0	4.69/4.67
	80S ribosome (4X down-sampling)	10	44/0	40.93/37.51
	TRPV1 (2X down-sampling)	10	44/0	14.12/13.33

Supplementary Table 2: The resolution of the final 3D map on three datasets

Algorithm	Dataset	Number of Classes (Original/with Preprocessing)	Resolution(A)@0.143 (Original/with Preprocessing)
CL2D	beta-galactosidase	64/64	8.60/7.67
	80S ribosome (4X down-sampling)	512/512	3.11/3.10
	TRPV1 (2X down-sampling)	128/128	3.26/3.21

Supplementary Table 3: The SNR of original dataset and the 2SDR de-noised dataset, the SNR is estimated using ASPIRE [11].

Dataset	SNR (Original/with 2SDR)
70S ribosome	0.019 / 0.805
beta-galactosidase	0.037 / 1.665
80S ribosome	0.010 / 3.317
TRPV1	0.003 / 2.717
NanoD-TRPV1	0.006 / 2.850

References

[1] Scheres, S. H. A Bayesian view on cryo-EM structure determination. *Journal ofMolecular Biology* **415**, 406–418 (2012).

[2] Sorzano, C. *et al.* A clustering approach to multireference alignment of single-particleprojections in electron microscopy. *Journal of Structural Biology* **171**, 197–206(2010).

[3] Liao, H. Y. & Frank, J. *Classification by bootstrapping in single particle methods* in*IEEE International Symposium on Biomedical Imaging: From Nano to Macro* (2010),169–172.

[4] Chen, T.-L. *et al.* γ-SUP: A Clustering Algorthm for Cryo-ElectronMicroscopy Images of Asymmetric Particles. *The Annals of Applied Statistics* **8**, 259–285 (2014).

[5] Elmlund, H., Elmlund, D. & Bengio, S. PRIME: probabilistic initial 3D model generation forsingle-particle cryo-electron microscopy. *Structure* **21**, 1299–1306 (2013).

[6] Scheres, S. H. *et al.* Disentangling conformational states of macromolecules in 3D-EMthrough likelihood optimization. *Nature Methods* **4**, 27 (2007).

[7] Elad, N., Clare, D. K., Saibil, H. R. & Orlova, E. V. Detection and separation of heterogeneity in-molecular complexes by statistical analysis of their two-dimensional projections. *Journalof Structural Biology* **162**, 108–120 (2008).

[8] Cong, Y., Kovacs, J. A. & Wriggers, W. D fast rotational matching for image processing ofbiophysical data. *Journal of Structural Biology* **144**, 51–60 (2003).

[9] Scheres, S. H. RELION: implementation of a Bayesian approach to cryo-EM structuredetermination. *Journal of Structural Biology* **180**, 519–530 (2012).

[10] Yang, Z., Fang, J., Chittuluru, J., Asturias, F. J. & Penczek, P. A. Iterative stable alignment andclustering of 2D transmission electron microscope images. *Structure* **20**,237–247 (2012).

[11] *ASPIRE* version 0.14.0.http://spr.math.princeton.edu/.
