# Supplementary material for: Pre-pro is a fast pre-processor for single-particle cryo-EM by enhancing 2D classification
Source: Commun Biol. 2020 Sep 11;3:508. doi: 10.1038/s42003-020-01229-0 (PMC7486923; doi:10.1038/s42003-020-01229-0)
Supplement: Supplementary file 2 — Description of Additional Supplementary Files [file 42003_2020_1229_MOESM2_ESM.pdf]

Description of Additional Supplementary Files

File Name: **Supplementary Data 1**
Description: source data for the graphs presented in the main figures
