# Supplementary material for: Pre-pro is a fast pre-processor for single-particle cryo-EM by enhancing 2D classification
Source: Commun Biol. 2020 Sep 11;3:508. doi: 10.1038/s42003-020-01229-0 (PMC7486923; doi:10.1038/s42003-020-01229-0)
Supplement: Supplementary file 3 — Reporting Summary [file 42003_2020_1229_MOESM3_ESM.pdf]

nature research

Corresponding author(s): Wei-Hau Chang

Last updated by author(s): Jun 24, 2020

Reporting Summary

Nature Research wishes to improve the reproducibility of the work that we publish. This form provides structure for consistency and transparency in reporting. For further information on Nature Research policies, see Authors & Referees and the Editorial Policy Checklist.

Statistics

For all statistical analyses, confirm that the following items are present in the figure legend, table legend, main text, or Methods section.

n/a	Confirmed	
☐	☒	The exact sample size (*n*) for each experimental group/condition, given as a discrete number and unit of measurement
☐	☒	A statement on whether measurements were taken from distinct samples or whether the same sample was measured repeatedly
☒	☐	The statistical test(s) used AND whether they are one- or two-sided<br>*Only common tests should be described solely by name; describe more complex techniques in the Methods section.*
☒	☐	A description of all covariates tested
☒	☐	A description of any assumptions or corrections, such as tests of normality and adjustment for multiple comparisons
☒	☐	A full description of the statistical parameters including central tendency (e.g. means) or other basic estimates (e.g. regression coefficient) AND variation (e.g. standard deviation) or associated estimates of uncertainty (e.g. confidence intervals)
☒	☐	For null hypothesis testing, the test statistic (e.g. *F*, *t*, *r*) with confidence intervals, effect sizes, degrees of freedom and *P* value noted<br>*Give P values as exact values whenever suitable.*
☒	☐	For Bayesian analysis, information on the choice of priors and Markov chain Monte Carlo settings
☒	☐	For hierarchical and complex designs, identification of the appropriate level for tests and full reporting of outcomes
☒	☐	Estimates of effect sizes (e.g. Cohen's *d*, Pearson's *r*), indicating how they were calculated

Our web collection on statistics for biologists contains articles on many of the points above.

Software and code

Policy information about availability of computer code

Data collection	No data was collected in house. The data collection scheme for the six used datasets is described in the Method section.
Data analysis	Pre-Pro is described in this work. 2D classification was performed with RELION 2.1 (Except NanoD-TRPV1 is performed with RELION 3.0), EMAN 2.21 and Xmipp3. 3D Reconstruction was conducted with PRIME 2.5 and cryoSPARC 0.65. For additional data analysis software was CTFFIND4, Scipion 1.2, UCSF Chimera 1.13 and Phenix 1.14.

For manuscripts utilizing custom algorithms or software that are central to the research but not yet described in published literature, software must be made available to editors/reviewers. We strongly encourage code deposition in a community repository (e.g. GitHub). See the Nature Research guidelines for submitting code & software for further information.

Data

Policy information about availability of data

All manuscripts must include a data availability statement. This statement should provide the following information, where applicable:

- Accession codes, unique identifiers, or web links for publicly available datasets
- A list of figures that have associated raw data
- A description of any restrictions on data availability

The 70S ribosome can be found in https://www3.mrc-lmb.cam.ac.uk/relion/index.php?title=Classification_example. The beta-galactosidase can be downloaded from RELION 2.1 tutorial data (https://drive.google.com/file/d/17e7MCk6-FP3X9Jn9-U-WvWw-L1oY178V/view?usp=sharing). 80S ribosome is from EMDB (accession number EMPIAR-10028, https://www.ebi.ac.uk/pdbe/emdb/empiar/entry/10028/). TRPV1 channel is available in EMDB (EMPIAR-10005, https://www.ebi.ac.uk/pdbe/emdb/empiar/entry/10005/). TRPV1 embedded in nanodisc is available in (EMPIAR-10059, https://www.ebi.ac.uk/pdbe/emdb/empiar/entry/10059/). The 3D maps in Figure 4 and 6 are available in https://drive.google.com/file/d/1iihyVw1Jy7ob9fKcq3ewkfZ8350qPFRM/view?usp=sharing.

Field-specific reporting

Please select the one below that is the best fit for your research. If you are not sure, read the appropriate sections before making your selection.

☒ Life sciences ☐ Behavioural & social sciences ☐ Ecological, evolutionary & environmental sciences

For a reference copy of the document with all sections, see nature.com/documents/nr-reporting-summary-flat.pdf

Life sciences study design

All studies must disclose on these points even when the disclosure is negative.

Sample size	The number of particles and class averages that went into 3D reconstruction is determined by each 2D classification algorithms with minimal human intervention (The good class average from RELION is selected according to statistics described in the Supplementary material).
Data exclusions	The data excluded from 3D reconstruction is again according to the criterion of each 2D classification algorithm as described in the manuscript.
Replication	Pre-Pro was replicated with six different cryoEM datasets and two or three state-of-the-art 2D classification algorithms. Experiments consistently report the benefits of using the pre-processor as discussed in the manuscript.
Randomization	During 3D refinements, data were randomly split into 2 groups following the "gold-standard Fourier shell correlation (FSC) procedure" (Sheres, 2012). In the pilot tests, the reference image is randomly selected.
Blinding	No blinding was conducted as data used is downloaded from previous analyzed benchmark dataset (EMDB and EMPIAR).

Reporting for specific materials, systems and methods

We require information from authors about some types of materials, experimental systems and methods used in many studies. Here, indicate whether each material, system or method listed is relevant to your study. If you are not sure if a list item applies to your research, read the appropriate section before selecting a response.

Materials & experimental systems

n/a	Involved in the study
☒	☐ Antibodies
☒	☐ Eukaryotic cell lines
☒	☐ Palaeontology
☒	☐ Animals and other organisms
☒	☐ Human research participants
☒	☐ Clinical data

Methods

n/a	Involved in the study
☒	☐ ChIP-seq
☒	☐ Flow cytometry
☒	☐ MRI-based neuroimaging
